# Exploring attitudes and preferences for dementia screening in Britain: contributions from carers and the general public

**DOI:** 10.1186/s12877-015-0100-6

**Published:** 2015-09-09

**Authors:** Steven Martin, Jane Fleming, Sarah Cullum, Tom Dening, Greta Rait, Chris Fox, Cornelius Katona, Carol Brayne, Louise Lafortune

**Affiliations:** Cambridge Institute of Public Health, University of Cambridge, Cambridge, UK; Academic Unit of Psychiatry, University of Bristol, Bristol, UK; Institute of Mental Health, University of Nottingham, Nottingham, UK; Research Department of Primary Care and Population Health, UCL Medical School, UCL, London, UK; Faculty of Medicine and Health Sciences, Norwich, UK Medical School, University of East Anglia, Norwich, UK; Division of Psychiatry, University College London, London, UK

## Abstract

**Background:**

Dementia is becoming one of the most important emerging public health concerns in a generation. In societal approaches to the mitigation of major disease ‘burden’, population screening can sometimes provide an effective approach to improving detection of disease and outcomes. However the acceptability of a systematic population screening programme for dementia, to the British public, is not known.

**Methods:**

A Patient and Public Involvement (PPI) event was organised to give members of the public from the East of England an opportunity to offer their perspectives and to comment on the findings of a systematic literature review looking at attitudes and preferences towards screening for dementia. The event was attended by 36 members of the public and eight national Alzheimer’s Society Research Network volunteers. The morning discussion contained a presentation, which defined population screening for attendees but contained no reference to the findings of the review. In the afternoon, findings of the review were presented and a discussion on the results was facilitated. The discussions were recorded, transcribed and subjected to thematic analysis. The NVivo qualitative data software was used to facilitate this process.

**Results:**

A total of 23 key themes emerged in relation to the carer and general population. The most frequent themes which emerged were the low levels of understanding and awareness around the dementia syndrome; the acceptability and validity of any tests; costs to the National Health Service (NHS); an individual’s existing health status existing health status; financial/profit motive for screening; the inability to change prognosis; and the importance and availability of support.

**Conclusions:**

Factors such as personal beliefs, experiences and attitudes to health impact on decisions to be screened for dementia. A number of additional concerns were raised which were not previously identified in the systematic literature review. These were around the economic incentives for screening (profit motive), the provision of social support, and the economic/social impacts of screening programmes. This may reflect cultural differences in health and social care funding models between Britain and other countries where previous research was conducted.

**Electronic supplementary material:**

The online version of this article (doi:10.1186/s12877-015-0100-6) contains supplementary material, which is available to authorized users.

## Background

Despite the best efforts of clinicians and researchers to improve the detection of dementia, many people still do not receive a formal diagnosis or receive it late in the disease progression [1]. To address this issue the EU joint action programme ALCOVE (Alzheimer’s Cooperative Valuation in Europe) is conducting research seeking to compare national recommendations for diagnosis and develop recommendations to “improve early diagnosis”. Screening has been proposed as one mechanism to improve both rates of diagnosis and patient care outcomes. In the UK doctors are recommended to “proactively” ask patients at risk of dementia about their memory, and offer a screening test [2]; however guidance produced by the UK National Screening Committee [3], the Royal Australian College of General Practitioners [4], and the US Preventative Services Task Force [5] advises against the adoption of screening for the early detection of dementia given the number of difficulties in implementing a screening programme [1, 4, 6], the lack of evidence that dementia fulfils established criteria to justify screening [3–5] or that such a screening programme would be acceptable to the clinicians, patients, carers or the public [7].

Our group has conducted a systematic review of research to date [7] on the attitudes and preferences of healthcare professionals, people with dementia, caregivers and members of the general public to dementia screening. The findings of the review suggest that screening for dementia raises complex issues around preference and choice for clinicians and the public: while some would favour a screening intervention, such an approach may not be amenable to all. However, the published literature on attitudes towards population screening for dementia is diverse and fragmented and it is unclear what specific factors promote or reduce screening acceptance the most therefore it is difficult to draw clear conclusions. Given the paucity of evidence on public opinion the acceptability of dementia screening is still unclear. A PPI (Patient and Public Involvement) event was organised to facilitate members of the public in the East of England to talk about their views on population screening for dementia. The aim was to contextualise the findings of a systematic review [7] for a British audience.

### Approach

The integration of PPI into public health research is becoming increasingly important in the UK. There is growing recognition that lay individuals need to be involved in the scientific process [8, 9]. It is believed that the involvement of lay members of the public may lead to the generation of new research questions or hypotheses, enhance the relevance and applicability of findings, and lead to the reprioritisation of research and policy.

## Methods

A PPI event was organised on the 5th June 2013. Delegates attending the event were viewed as partners rather than research subjects in order to generate data and evidence collectively. In this exercise, The National Coordinating Centre for Public Engagement definition was used; “Public engagement describes the myriad of ways in which the activity and benefits of higher education and research can be shared with the public. Engagement is by definition a two-way process, involving interaction and listening, with the goal of generating mutual benefit” [10]. This PPI exercise is based on the reporting standards set forward in the GRIPP (Guidance for Reporting Involvement of Patients and Public) checklist [11].

### Description of involvement activity

Members of the public were invited to attend a one day event held in Cambridge City (UK) by a firm experienced in recruiting (detail presented below). The event was jointly organised by the Alzheimer’s Society (East and London Regions) and the University of Cambridge. The PPI event was a consultation during the final stages of the systematic reviewing process. The morning discussion was framed around a general presentation conducted by a member of the research team (LL) and undertaken to define screening. It contained no reference to the findings of the systematic review. In the afternoon the aims of the review, methodology, analysis strategy, and findings were presented. The focus of this PPI event was on the screening of asymptomatic individuals using paper and pencil tests. A discussion (in groups of no more than 12 people) on the meaning of those findings for attendees was facilitated. Attendees were divided into broadly age-banded groups with the ASRN (Alzheimer’s Society Research Network) members in a separate group from the wider public groups. The ASRN is constituted of volunteers, usually people with dementia or their carers.

### Recruitment of partners

General public: The research team considered what the target audience’s specific needs may be, in terms of both the best approach to recruitment and the best approach to getting people engaged in a project and approached a private firm to undertake recruitment. A purposive approach was adopted to ensure engagement with a cross-section of the public in the locations selected. The University of Cambridge provided a sample specification together with a screening letter and consent form. We would try to recruit a representative sample based on the 2011 census for Cambridge, England (16.4 % aged 65 and over; 50 % for both male and female; 60 % in an occupation). Adherence to these demographics was not strict. The recruiters then used a range of methods for free-finding participants including on-street recruitment, approaching areas where the target audience is more likely to visit, for example if the target was older people they could work with local services providing facilities for older people. Strict protocols were in place for managing personal data. All partners signed a consent form before the event, at the point of recruitment the recruiter asked the respondent to read and sign the form. Forms were then returned to the University of Cambridge via special delivery. Any partners that were missed were then contacted and consent provided via email. Respondents were also asked about special dietary requirements and mobility issues.

Alzheimer’s Society group: The ASRN recruited members of this group. An initial e-mail was sent to Eastern region members to ascertain initial interest. Those interested in participating indicated by email and were subsequently provided with the dates, times and venue of the event. A consent form was signed before the event and returned to the University of Cambridge.

### Description of partners

A total of 50 people were invited to participate in the PPI event (Table 1). The event was well attended, with 36 members of the general public from across Cambridgeshire and eight ASRN volunteers. No one attending the event identified themselves as having dementia, however, eight people in attendance had a member of their family with dementia, ten had cared for someone with dementia and five worked with people with dementia, eleven reported they had little or no direct experience of dementia. The PPI event involved two ASRN monitors in the management committee; both had a role in the development and preparation leading to the event but did not participate in the group discussions.

**Table 1:** Participants at PPI event

Female	*n* = 25 (50 %)
Male	*n* = 19 (38 %)
Withheld information	*n* = 6 (12 %)
Aged under 35	*n* = 11 (22 %)
Aged 35–44	*n* = 6 (12 %)
Aged 45–54	*n* = 6 (12 %)
Aged 55–64	*n* = 11 (22 %)
Aged 65–74	*n* = 7 (14 %)
Aged over 75	*n* = 5 (10 %)
Withheld information	*n* = 4 (8 %)

### Detail of discussion

A quasi-focus group format was used to collect the perspectives from those present. There was no interview schedule for the discussion groups; the round table debates were based on the issues raised by our partners and the review. The comments of those attending the event were recorded by a facilitator on a flip chart (and electronically) and subsequently reported back once all participants had reconvened. The discussions were then transcribed and subjected to thematic analysis using the NVivo qualitative data software package. Members of the research team (LL and SM) made themselves available to speak with attendees after the event to answer any remaining questions, or to provide clarification or advice.

### Questionnaires

Questionnaires were handed to partners attending the event at the beginning and end of the day. These questionnaires collected data on attitudes and preferences to screening for dementia, and were undertaken to examine the degree to which attitudes and preferences changed during the event (Table 2).

**Table 2:** Pre/post screening questionnaire results

Question	Before	After
I think I have more problems with my memory than others my age	72 % no	75 % no
	13 % yes	15 % yes
	(6 missing item response)	(4 missing item response)
Has a doctor told you that you have a problem with your memory	88 % no	88 % no
	12 % yes	12 % yes
	(4 missing item response)	(4 missing item response)
I would like to know if I have a problem with my memory	13 % strongly agreed	13 % strongly agreed
	34 % agreed	36 % agreed
	25 % unsure	18 % unsure
	11 % disagreed	18 % disagreed
	6 % strongly disagreed	24 % strongly disagreed
	(4 missing item response)	(4 missing item response)
I would like to know if I am more likely than others to have dementia	11 % strongly agreed	13 % strongly agreed
	34 % agreed	36 % agreed
	27 % unsure	18 % unsure
	15 % disagreed	18 % disagreed
	12 % strongly disagreed	24 % strongly disagreed
	(4 missing item response)	(4 missing item response)
I would like to know I have dementia	13 % strongly agreed	11 % strongly agreed
	40 % agreed	34 % agreed
	20 % unsure	13 % unsure
	11 % disagreed	25 % disagreed
	24 % strongly disagreed	24 % strongly disagreed
	(4 missing item response)	(4 missing item response)
How often would you like to be tested after	5 people said yearly	
	4 people said every 2 years	
	3 people said every 5 years	
	1 person said once a decade	
	4 people said they didn’t know	
	6 people said never	
	11 people made no response	
	1 person said from age 45 - every 2 years	
	1 person said until scientifically proven	
	1 person said depends on test	
	1 person said if symptoms present	
	3 people said as required	
	1 person said Memory test. Biomarkers	
People should be tested for dementia	18 % strongly agree	20 % strongly agree
	50 % agree	20 % agree
	15 % unsure	18 % unsure
	1 disagree	15 % disagree
	2 strongly disagree	11 % strongly disagree
	(4 people withheld information)	(4 people withheld information)
People should be tested for colon cancer	25 % strongly agree	29 % strongly agree
	56 % agree	43 % agree
	3 unsure	9 % unsure
	No one disagreed	6 % disagree
	1 strongly disagree	No one strongly disagreed
	(4 people withheld information)	(4 people withheld information)
People should be tested for depression	18 % strongly agree	25 % strongly agree
	47 % agree	25 % agree
	20 % unsure	22 % unsure
	1 disagree	15 % disagree
	No one strongly disagreed	No one strongly disagreed
	(4 people withheld information)	(4 people withheld information)
Being tested for dementia can cause no harm	15 % strongly agree	18 % strongly agree
	25 % agree	9 % agree
	38 % unsure	29 % unsure
	1 disagree	20 % disagree
	9 % strongly disagree	11 % strongly disagree
	(4 people withheld information)	(4 people withheld information)
I didn’t find these questions difficult (NB: People found this question ambiguous)	38 % strongly agree	29 % strongly agree
	45 % agree	43 % agree
	1 unsure	2 unsure
	1 disagree	3 disagree
	1 strongly disagree	1 strongly disagree
	(4 people withheld information)	(6 people withheld information)

### Economic appraisal

In recognition of participants’ time and to mitigate the risk of participant dropout, a fixed monetary honorarium of £80 was offered and reminder calls were made in advance of the event. Costs for supportive care were provided to partners if they attended without their care recipients, and travel costs. The organisational costs (including food and venue) reached a total of £1206. Recruitment and payment of partners and administrative support for the event cost a total of £4,075. Stationary and printing cost a total of £40, while money for ASRN participants was a total of £480. Other costs included £255 for transcription of recordings. In total the cost of the public event was £6,056.

### Analysis

Thematic analysis was undertaken independently by two researchers (SM and LL). Two reviewers worked independently and then compared findings to produce a mutually agreed coding framework; also informed by a previous systematic review [7]. Statements related to the partner’s perceptions, views and/or attitudes and/or experiences, ethical, moral and cultural opinions were coded and analysed in NVivo. Data were synthesised and themes were agreed. Rater discrepancies were resolved through discussion. We then analysed the nature of the evidence on attitudes and preferences of three key time periods of screening: (1) pre-screening, (2) in-screening and (3) post-screening periods. Summaries were sent to PPI partners to read and critique. By involving our PPI partners in the analysis we were able to have members of the general public inform our interpretation and synthesis of the data. While the authors retained editorial control over the analysis, considerable weight was given to their comments. Partners’ responses to the summaries were confirmatory of the authors’ data interpretation. Descriptive analysis was undertaken on the questionnaire data.

## Results

### Context

Findings from a systematic review of attitudes and preferences towards screening for dementia [7] were largely drawn from studies undertaken in non-UK healthcare contexts. Both the research team and the funders, a major UK dementia charity, felt it important that the review be contextualised to better understand the finding’s relevance to the UK and enhance the review’s impact.

### Themes

**Fig. 1:**
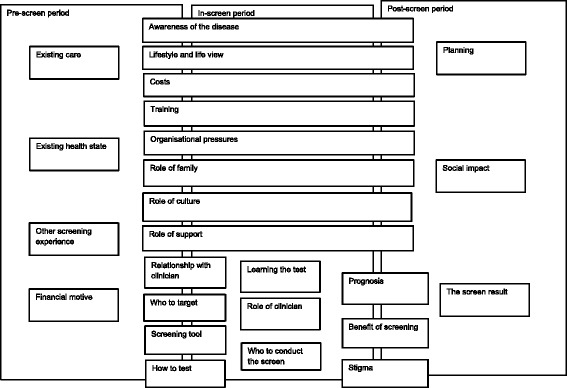
Model of themes identified

From the discussions at the event 23 key themes emerged. A framework was developed that divided the screening process into 3 stages: (1) pre-screening, (2) in-screening and (3) post-screening periods (Fig. 1). *Pre-screen:* existing care, existing health status and other screening experience. *In-screen:* the screening tool, role of clinician, how to test, learning the test, who to conduct the screen, relationship to doctor and awareness of the disease. *Post-screen:* social impact, the screen result, prognosis and stigma. Many of the themes were influenced by the individual’s social context such as; lifestyle and life view, role of family, role of support. Also the wider health and social care organisational context such as financial motives, costs, on whom to target the screening test, organisational pressures, and planning.

Themes only raised by the ASRN group included the relationship to the doctor, organisational pressures and the social impact of dementia screening. Themes exclusive to the general public group were training for staff, and the role of the family (Table 3). A summary of themes can be found in Table 4; a comprehensive table is provided as Additional file 1: Table S1.

**Table 3:** Overlapping themes at PPI event

Themes exclusive to Alzheimer’s group		Themes exclusive to general public	
Relationship to doctor	Organisational pressures	Provision of information	Role of culture
Social impact		Training for staff	
**Themes which crossed boundaries**			
Who to target	Role of support	Stigma	Who should conduct the screen
Suitability of the test	Costs	Lack of ability to change prognosis	Financial motive
Role of planning	Existing health	Acceptability of test	Level of screen inaccuracy
Awareness of disease	Existing care	Role of family

**Table 4:** Themes identified in studies

Pre-screen	
Theme	Evidence
Accuracy of the test	“I’d want to know how effective it is… I’d want to be given some figures”; “Yeah, the accuracy of the test is everything. At the moment it’s our considered opinion that the test is in its infancy”.
	“But what are you going to screen for? In bowel screening and in breast screening it’s quite clearly known what you’re searching for and it’s quite clearly known what you’re going to do if you find it. My first question is what are you going to screen for?”
Existing care	“A lot of the staff members… treated them (patients) like animals”.
	“I mean if you are in the system already you’re much more likely to see the publicity about do you want to join… you know, sign up in the thing, you know, you’re more… or you’re in the hospital you see a sign and think oh yes… perhaps I should be doing that!”
Existing health state	“You’ve already been diagnosed with something and they’re helping, and you’re being treated, so there is a cause and an answer which would create more confidence”.
	“Worry more”.
Experience with disease	There was no consensus between those with experience of the disease and those without. Some, with experience, argued, “I have a few acquaintances who have dementia, and it makes it much more real”. These were more likely to accept screening. However, some had family members with the disease and were dissuaded from undertaking the screen because of this experience: “Having someone in the family or close with a condition does not make you any more likely to seek answers for it”.
	Some argued that previous experience had no bearing on what is perceived as an individual choice: “Having someone in the family, I wouldn’t say affects you at all, affects your likelihood of going for screening at all”.
Financial motive	“Somebody could make money”;
	“I would have suspicions if it was being done by a drug company”.
Lifestyle and life view	“It’s the balance… of what you have to go through for the screening… what the benefits are, you get a return, or carers get a return, it’s that sort of balance”.
	“Yeah, exactly, it’s a personal choice, and I think if you know that your lifestyle could lead to dementia you’d probably be more comfortable with your lifestyle and not want to worry about dementia.”
Other screening experience	“Is very invasive of your body”.
	“That’s completely different, the way you think about it and anticipate it”.
Role of clinician	“My daughter (is) qualified (as doctor), she was treating people… going to discharge them and saying has their dementia been managed… and consultants were saying to her ‘Well, they’re not diagnosed with dementia’… She’s always trying to get them care plans and fighting for their support, but you know, while it’s not even known widely at that level amongst the professionals, then I think there’s a lot of work to be done, and not just in the community”.
Role of culture	“My wife is Chilean, and when we go over there it is a family concern over there, they really do look after each other”.
	“I live in the Philippines most of the year and I have a big extended family, and they look after me… and it is one advantage you have in third world countries, they have extended families… there is no suffering”.
Role of family	“A lot of people tend to keep it in the family, than let outsiders know that it is happening, don’t they? That is the problem.”
	“It sounds dreadful but in fact, it is true isn’t it, for a family to have to learn that some member of the family is going to come down… be felled by this disease is just awful. You’re living on a knife edge and borrowed time”.
Who to target?	“School”.
	“You should target everyone rather than a certain group of people like based on age or ethnicity or experience or whatever”.
In-screen	
Theme	Evidence
How to test	“Would brain scans be appropriate for a screening”.
	“The form of screening that I would opt for, if there was a choice, would be to go and have the head scan because it shows whether you’ve got Parkinson’s or Alzheimer’s. I mean, I do know that because that’s how we found out my mother had got it, and my uncle, and what they’d got, because green, I think it’s green, or was it blue, is the Alzheimer’s, red is the Parkinson’s”.
Learning of test	“Yeah, the woman actually went in there and she did a test, a memory test with my mother, and my mother already previously knew that this woman was coming because they have to tell them, and I went, and I was sitting there and I’m thinking… well, you’re asking her questions that she’s already logged in her head and she knows, and she’s going to reel them off, as she was doing”.
Organisational pressures	“The doctor doesn’t have time, you know, you rush in, you’ve got five minutes, and away you go, so if you have two ailments and you have two questions you have to get another appointment, so you know, why is this doctor doing the screening?”
	“It would put more pressure on the GPs because they would have to have training, which means more closed surgery days, and they will have to have special times to do that, you can’t do it on a normal ten minute appointment. So yeah, that would put pressure on them to be honest”.
Relationship to doctor and health	“I think if you’ve got a good rapport with your doctor, you feel comfortable, and probably with a doctor you’re familiar with, they spend a bit more time with you than a doctor you don’t know, so I think that probably… well, in my case it would make it easier”.
	“Even if you just visit regularly for different things, and if you have the same GP you see all the time, if they come and suggest this screening… you might be more inclined to accept it. Because otherwise, if you see a different GP every time and one of them offers it to you, you say no, I’ll pass. I think that, yeah”.
Training needs for staff	“Well they need to be trained, it’s got to be someone who’s trained… and it’s not going to be achieved”.
	“It’s got to be somebody trained to do that and not anything else”.
	“Yes, we’re looking for the good test that a trained person can administer”.
Who should conduct the screen?	“I think as a general practitioner they get five minutes with you, and to make decisions you know… so maybe you’re better off with a more specialist… somebody who deals with this”.
	“A carer that does a test”.
	“Family, carers, nurses”.
Post-screen	
Theme	Evidence
Planning	“Rather know to be prepared, you know, set things in place before you are in a state where you can’t remember anything”.
	“Don’t know whether I’m going to end up in a wheelchair or not”.
Role of support	“Getting them involved in providing a good support network for each other, to be there for each other as well as the person affected, and I think it probably just wakens people up to just sort of try and have a better quality of life rather than dwell on problems and depression and bad weather”.
	“Screening cannot interfere with my life. As a woman I have many different roles in my home, looking at my family and husband and depending on what is happening, I might not have time to go for screening”.
Social impact	“One of the things that would put me off I think is like… maybe you test positive… what if that (information) gets into the wrong hands, like employers or like… that can affect you too, like ‘Oh well we won’t employ that group because…’ Someone’s got information somewhere about you which is possibly… that’s what would put me off, that would be a negative”.
	“But if you’re going to risk losing your job, if you’re going to risk losing whatever in your current lifestyle, then you’ll probably back off until there is a precise treatment”.
The screen result	“I am not sure knowing actually benefits me”.
	“If you’re the kind of person who hides from the truth and reality, you’re not going to be any more likely to go”.
	“I’d definitely want to know. I’ve seen it with my father, and my mother trying to hide it from everybody, she was frightened to death of what people would say, and even neighbours she tried to hide it from them, and you know it was ridiculous really, but she just wouldn’t get advice or help: ‘I can cope, I can cope’”.
	Themes which cut across the pre-, in-, and post-screen process
Acceptance of test	
“I wouldn’t want to be screened… if there was any side effects or if the test got too intrusive”.	
“If you said a simple written test, it’s not a simple written test for anyone who can’t write… so a simple written test will eliminate quite a fair number of people”.	
Awareness of disease	
“Alzheimer’s is one of these nebulous, I am not really sure what it is”.	
“Alzheimer’s… there is somewhere around 100 different forms of Alzheimer’s”.	
Costs	“If there is no positive implications (no cure), is it worth spending the money”; “That’s an awful lot of money in terms of paying the doctors and time taken off their work”; “It would probably be very expensive”; “It could be better spent”; “Well it’s a waste of money”; “There is no funding for this”; “I mean unless they’re going to put money into the system it’s actually fairly pointless”.
	“There’s more and more money being taken out of the NHS, and this system, screening, is requiring more money not fewer doctors and nurses and care workers. So why… forget it, put the money into research. Forget the screening”.
Lack of ability to change prognosis	“I’d still rather be in my house where I’ve got a familiar environment to me, and I know where… especially if I know my brain is deteriorating, the last thing I’d want is to be surrounded by strangers in a completely different environment and everything’s in a set regime and all that sort of stuff. So I think yeah, a care home is probably the last place I’d want to be”.
	“I’d probably change my opinion on it if I knew for certain there was actually a treatment for it that worked. But at the moment I just think there’s so much research, but there’s no treatment for it”; “I think you’re better not knowing nothing about it at all, personally speaking I’d rather not know at all, but I… I could be influenced”.
Patient Benefit	“Dementia, it’s one of those ones where if you get it there’s not much you can do about it, so unless I had specific reasons to do it I don’t think I’d have it”
	“(They) see it as quite positive really, I think I am lucky to live in a country where there is this sort of screen for this disease”.
Stigma	“I think there’s a sort of stigma attached to going for screening, and people will be ducking and diving, you know, so I think it’s important”; “If you are not careful you are going to be labelled”; “Because of the stigma”; “Rightly or wrongly, it has a stigma”.
	“People (would be) judging you for an illness (and this) shouldn’t really be right”; “Too many of the population have a stigma, you know, and… just because they don’t know how to handle it in my view”.

### Pre-screen

#### 1. Existing care

Delegates with existing care needs mentioned that their current health was an important factor when deciding if they would attend for a screening test: *“I mean if you are in the system […] or you’re in the hospital, you see a sign and think ‘Oh yes […] perhaps I should be doing that!”* Conversely, experience of poor quality care makes individuals less likely to attend screening. Attendees who had witnessed poor care also indicated that this was an additional factor that would make them less likely to attend.

#### 2. Existing health status

There was no consensus on the impact of an individual’s existing health status towards a decision to screen. While most thought that an awareness of conditions would result in improved care *“You’ve already been diagnosed with something and they’re helping […] which would create more confidence”*. Others argued that knowing would make individuals “*worry more*”. There was a high level of variation between responses.

#### 3. Other screening experience

Experiences of other screening tests and programmes were discussed, most commonly breast and cervical cancer screening. Screening for dementia however; *“that’s completely different, the way you think about it and anticipate it”*. Previous screening experience was seen as relevant but how far such experience or lack of experience might affect individual decision-making was unclear.

### In-screen

#### 1. The screening tool

Attendees had a number of concerns regarding test accuracy. Many wanted to have evidence that the test works effectively before undertaking a screen; *“I’d want to know how effective it is […] I’d want to be given some figures”; “Yeah, the accuracy of the test is everything”.* Concerns were also raised around pen and paper tests, which may not be understood by everyone, given language and cultural differences. Where partners had experience of being tested for dementia they noted the Mini-Mental State Examination as being a particularly “stressful” tool.

#### 2. Role of the clinician

Some delegates argued that there is insufficient agreement on screening within the health profession to make it a viable programme *“(My) daughter (is) qualified (as a doctor) and she was treating people […] and saying has their dementia been managed […] and consultants were saying to her well, they’re not diagnosed with dementia […] She’s always trying to get them care plans and fighting for their support, but […] I think there’s a lot of work to be done, and not just in the community”.*

#### 3. How to test

There was no consensus on how a screen should be undertaken. The idea of computerised or web-based screening was popular, as was brain imaging and genetic testing; *“Would brain scans be appropriate for a screening?”; “The form of screening that I would opt for [..] would be to go and have the head scan*”. Delegates were reminded that pen and paper tests were the focus of the review [7], but conversations repeatedly returned to brain scans and genetic testing, so there is insufficient evidence to draw any robust conclusions on the attitudes and preferences towards simpler screening tests.

#### 4. Learning the test (repeated test exposure)

For those delegates who did discuss the use of pen and paper based screening tests, there were some concerns that the test itself may become less effective over time *“Yeah, the woman actually went in there and she did a test* […] *and I’m thinking […] ‘Well, you’re asking her questions that she’s already logged in her head and she knows, and she’s going to reel them off’, as she was doing”*.

#### 5. Who should conduct the screening test?

There was no strong preference for a particular healthcare professional to administer the screen; however delegates agreed that it should be a trained individual. The most frequently cited examples were the patient’s owns doctor, nurse, or a memory specialist: *“I would prefer a doctor, my own GP”; “It could be a memory clinic […] it could be a department of the hospital”; “Well, have mobile vans coming round!”; “Social Services”; “Family, carers, nurses”.*

#### 6. Relationship to doctor & health care system

The relationship to the doctor was seen as central to the decision to screen *“I think if you’ve got a good rapport with your doctor* […] *it would make it easier”; “Even if you just visit regularly for different things,* […] *you might be more inclined to accept it”.* For some attendees the relationship was correlated with the existence of comorbidities; *“I mean if you have two illnesses you’re much more likely to have a rapport with your clinician aren’t you?”.*

#### 7. Awareness of the disease

Awareness of dementia could both increase and decrease the likelihood of attending dementia screening. Some delegates with experience of caring argued that those with *“acquaintances who have dementia”* were more likely to accept screening because it “*makes it much more real”*; however, some carers were unenthusiastic: *“having someone in the family or close with a condition does not make you any more likely to seek answers for it”.* Some argued that previous experience had no bearing on what is perceived as an individual choice: *“having someone in the family I wouldn’t say […] affects your likelihood of going for screening at all”.*

There was some uncertainty over the syndrome, how it would impact on their life and on their family, friends and social contacts *“Alzheimer's is one of these nebulous […] I am not really sure what it is”; “how far (has) science or knowledge has gone with regards to understanding Alzheimer’s or dementia. Are they two words for the same thing?”* There were also some uncertainty over the cause of illness*: “Maybe it could be down to stress […] I don’t know […] fertilizer they put on food”*; *“Some say it’s the scrapings off the pot, when you’re cooking using a grey aluminium pot”.*

### Post-screen

#### 1. Social impact

There were a number of concerns about the societal impact of screening *“I think all that might do is just engender tremendous anxiety in the population at large”; “I think we think it could be detrimental and it also […] it’s unnecessary”.* There were confidentiality concerns that the screen result would be disseminated wider than healthcare professionals*: “One of the things that would put me off I think is […] What if that (information) gets into the wrong hands, like employers […] that would be a negative”.*

#### 2. The screen result

There was wide-ranging discussion of potential reactions to receiving the screening result. Whilst some delegates would want to know their results *“You should find out for the sake of it”; “(if I knew) I wouldn’t have the stress of worrying about it”,* others preferred not to know. For some it was like being *“given a death sentence”*; *“It’s you slowly drown into pretty much a vegetable”.* Another responded *“I mean, excuse my bluntness, but if someone finds out […] that you will be completely forgetful and a drooling mess […] and we don’t want to be that”.* Some comments were more neutral *“from what we know right now … I am not sure knowing actually benefits me”.* One delegate said *“If I discover I have dementia then Jesus! My whole life and perception will change and try and shape things the other way around.”*

#### 3. Lack of effective treatment and prognosis

The unavailability of a cure was a concern for a large number of delegates and appears to have an impact on screening decisions. One noted: *“You’re still facing the possibility of being told that you might have a disease for which there is no cure”; “dementia, it’s one of those ones where if you get it there’s not much you can do about it”.* For some they would only attend a screen if the disease could be cured: *“If there was a cure or they could head off that disease that would be something to encourage you for it, for a test”.* While the lack of an available cure is a significant concern, there were also fears that existing treatments are inadequate. Importantly, some people expressed the view that their attitudes may be subject to change: *“I’d probably change my opinion on it if I knew for certain there was actually a treatment for it that worked.”*

Few people in the ASRN group saw a benefit to screening in the current context. The overall mood was characterised by a form of fatalism *“You can’t change what’s going to happen and what’s not going to happen”.* One delegate felt these views were not necessarily an argument against offering screening: *“it is my choice, so I would be informed about it beforehand and then I would make my choice, so I don't see a problem there”.*

#### 4. Stigma

For some attendees the perceived stigma of undertaking a screen test had a negative impact on their willingness to be screened: *“I think there’s a sort of stigma attached to going for screening”*; *“If you are not careful you are going to be labelled”; “…because of the stigma”; “Rightly or wrongly it has a stigma”.* There was a belief that *“people (would be) judging you for an illness (and this) shouldn’t really be right”; “Too many of the population have a stigma […] just because they don’t know how to handle it”.* One delegate got particularly emotional on this issue *“I’ve been a carer, and you have 10 min with that patient, they’re a human being for God’s sake”;* however, it was also argued that the level of stigma encountered by those with dementia is reducing across the society: *“mental health and everything […] now it’s not so much stigmatised, you know”.*

### Cross-cutting themes

#### 1. Lifestyle and life view

Lifestyle and life view were also important determinants, both the perception that certain lifestyles might affect dementia risk and the anticipation of lifestyle changes: *“If you’re going to risk losing your job […] losing whatever […] then you’ll probably back off until there is a precise treatment”*; *“I think if you know that your lifestyle could lead to dementia you’d probably […] not want to worry about dementia”*. Personal circumstances, such as having children or other dependents could affect decision-making. Mostly those with young children felt a responsibility to be screened, but others said it was more important for them to “*get on with family life*”.

#### 2. Role of family

The family plays a complex role in determining an individual’s decision to attend a screen for dementia. Families may disregard someone’s own concerns; one delegate mentioned a friend whose *“husband refused to take her”* to the doctors. Adding that the experience *“was upsetting and worrying for her […] it was terribly distressing”.* Families may also keep a dementia diagnosis to themselves *“A lot of people tend to keep it in the family, [rather] than let outsiders know that it is happening, don't they”.* Some delegates spoke about a shared experience *“It sounds dreadful, but in fact it is true, isn’t it, for a family […] You’re living on a knife edge and borrowed time”*. Issues around having a family and being female were also raised *“screening cannot interfere with my life, as a woman I have many different roles in my home, looking at my family and husband and depending on what is happening, I might not have time to go for screening”.*

Delegates from black and minority ethnic communities spoke about their cultural differences, in particular the role of the extended family which was seen as beneficial *“it is not a problem, because you have people around”; “one advantage you have in third world countries, they have extended families […]. There is no suffering”.* They spoke about the divisions of labour, the delegation of jobs and caring responsibilities within the family and home environment *“when we go over there it is a family concern over there, they really do look after each other”.*

#### 3. Role of support

Availability of support was one of the main themes emerging from all groups; *“You can’t cure it, but if there’d have been some help”; “I would want to live my life and not to worry, but if I was diagnosed with dementia and I had the right support around me, fair enough, I’d like to do things”; “getting them involved in providing a good support network for each other, to be there for each other as well as the person affected”.* It was also recognised that the provision of support was not related to the provision of screening *“you can get all of that without screening”.*

#### 4. Financial motive

A financial motive for screening was discussed in seven of the eight groups. There was a large degree of scepticism about the reasons screening was being discussed: “*somebody could make money*”. A number of attendees believed that the adoption of dementia screening was to improve their GPs’ *“pay scale”*. Pharmaceutical companies in particular were believed to be behind the drive for screening aiming for a further *“push on drugs”*. Many delegates mentioned that they “*would have suspicions if it (the screening) was being done by a drug company*”. Some delegates argued that “*the media [are] selling it (dementia) as a fear tactic*”. There was also a fear that insurance companies would also abuse the screen results, mentioning concerns that, if screened, people *“risked”* being “*clobbered for higher insurance*”. There was general agreed scepticism over the profit motive in provision of other care services, that seemed to colour views on postulated economic drivers for screening*, “now it is done on a commercial basis, on commercial terms, which are getting a profit out of it”.*

#### 5. Who to target

There was no agreement on who should be invited for dementia screening, and little awareness of the factors that might determine why a screening programme might target certain groups. No discussion groups raised relevant questions such as "When does dementia become more common?" or "Is there any evidence that can show how well these tests identify dementia at different ages?" One person noted that “*You should target everyone rather than a certain group of people*”. The youngest group which was recommended as a target was “*school age*” children, others suggested “*40 and upwards*”. While older age is a risk factor for dementia very few delegates identified this population as the target for any screening intervention. The exceptions were some of the ASRN delegates who were happy with the age specifications set in quality standards such as the Commissioning for Quality and Innovation targets set by the UK Department of Health [1].

#### 6. Organisational pressures

There were a number of concerns from delegates regarding the logistics of offering screening through general practice, mostly from an organisational and training perspective: “*The doctor doesn’t have time. You know, you rush in, you’ve got 5 min, and away you go*”. Another delegate noted “*It would put more pressure on the GPs because they would have to have training […] you can’t do it on a normal 10 min appointment”*.

#### 7. Training needs

There was a concern that with any new programme there are new skill requirements “*which means more closed surgery days”*. The groups did reach a consensus and indicated that the individuals administering the screen needed the adequate training to do so; no one was willing to be screened by untrained individuals. There was a significant concern that unqualified and untrained individuals may make unacceptable errors resulting in additional concern for the patients *“It’s got to be somebody trained to do that and not anything else”*.

#### 8. Planning

The need for planning after the test was raised in a number of groups. Some would *“set things in place before you are in a state where you can’t remember anything.”* For these participants the opportunity to make legal arrangements, such as a lasting power of attorney, was very important; this was raised in 3 of the 8 groups. Time to make other preparations, not necessarily dementia-specific, came up too; one delegate said that they had already started changing furniture and preparing their home for old age *“I don’t know whether I’m going to end up in a wheelchair or not”*. Conversely, some would not attempt to plan for the future; *“for me [it’s] not to do with jobs or insurance…just lifestyle “.* And some would not want to try to change their lifestyle, *“You can't be cured anyway, my guess is. Yes, have another glass and forget it all”.*

#### 9. Costs

For most delegates cost was a major factor; *“If there is no positive implications, is it worth spending the money?”; “That’s an awful lot of money in terms of paying the doctors and time taken off their work”; “It could be better spent”; “Well, it’s a waste of money”; “There is no funding for this”. “There’s more and more money being taken out of the NHS […] forget it, put the money into research. Forget the screening”.* Delegates from the ASRN had very strong preferences not to spend money on screening but on "*research*".

### Questionnaire results

Responses to the pre-post questionnaire demonstrated that a number of delegates changed their minds regarding their attitudes towards screening, many also changing their opinion on the benefits of population screening. From changes in questionnaire responses we can see that by the end of the event:

- Fewer delegates said they would like to know if they had a problem with their memory
- Fewer delegates said they would like to know they had greater risk of dementia
- Fewer delegates said they would like to know if they had a problem with dementia
- Fewer delegates said people should be tested for dementia
- Fewer delegates said they thought screening was harmless

Thirty delegates responded to the question “How often do you think individuals should be screened for dementia?” and answers varied widely; some partners responded that screening should be conducted on a yearly basis; others argued that it could be once a decade or never. Eleven people made no response to this question, four people said they didn’t know and six people said never.

### Process

There are a number of factors which impact on findings. Firstly, by only recruiting partners from the East of England and London regions we were unable to capture a diverse range of attitudes from across the country, meaning any variation at regional or national (Wales and England) level may be missed. Secondly, as some attendees volunteered via the ASRN, there is a potential source of bias as those with the strongest opinions and/or experiences may have attended the event thereby skewing the feedback.

### Impacts and outcomes

Findings from this public event provide some evidence that issues related to test accuracy, existing care, lifestyle and life view, organisational pressures, planning and the role of support that were identified in our international review are transferrable to UK health care. However, the magnitude these factors determine an individual’s willingness to be screened remains unknown due to cross-cultural sensitivities.

### Economic appraisal

As discussed the total cost of this PPI event was £6,055.80. For this amount we were able to contextualise findings from international research to a UK healthcare setting and inform a sample of 50 members of the public and carers about what a screening programme for dementia might involve; its benefits and drawbacks. While there were a number of positive outcomes from the event, there are also a number of difficulties that may impact on the ability to undertake PPI events in the future. The main issues include the use of resources, in particular staff time and costs. Timing was also an issue; it is important to ensure that both location and date is suitable for members of the general public. Recruitment was also a lengthy process as we were unsure how representative the group would be, which groups were required and how many delegates there needed to be. In the future researchers and funders need to make appropriate allowance for the considerable costs of PPI; larger budgets for supportive care costs may be allocated in order to improve the response rate from people with dementia themselves. There also needs to be better information about the availability of re-imbursement for costs of alternative care provision.

## Discussion

The main findings of this PPI exercise suggest the acceptability of screening is dependent upon a variety of factors including personal beliefs, experiences and attitudes to health. The most frequent themes that emerged were the awareness of the disease and its implications; the acceptability and validity of the test; the costs to NHS; existing health status; financial/profit motive; and the importance of support. While individual lifestyle factors are important, the decision to screen is inextricably linked to the lack of ability to change dementia prognosis. Although all involved consider dementia a serious condition, many questioned the need to identify it early through population screening.

There are a number of strengths to this PPI exercise, including the use of inclusive sampling methods to increase the representativeness of partners. The format facilitated discussions on those issues arising from the review, and topics were examined in depth. The inclusion of lay participants in the analysis and the writing of this article was also helpful because it allowed for the research team to confirm their interpretation of the discussion.

There are however a number of weaknesses. PPI is not research and these findings should not be seen as a replacement for robust qualitative inquiry. The pre-post questionnaire was not powered or designed to demonstrate statistical significance. The small number of partners attending the event also limits the generalizability of findings. Results are therefore descriptive and while findings are transferable to other settings further research would be required to understand which are most appropriate. The study also recruited a large proportion of partners who had worked with or cared for a person with dementia, which may contribute to a recruitment and selection bias and therefore may subsequently influence the representativeness of the findings. Problematically, there is a lack of large-scale mixed-method studies seeking to understand the social acceptability and implications of dementia screening; future studies could assess attitudes and preferences through a combination of interview and survey. Also, pen and paper tests were the focus of this project. Attitudes towards other means of screening for dementia, namely genetic screening, are likely to be driven by different factors. A systematic review specifically looking at these factors is underway [12].

In comparison with findings from our review of international research, there were greater concerns around the economic incentives around screening, the provision of social support, and the economic or social impacts of screening programmes. This may reflect cultural differences between the British population and other countries such as the USA which have adopted differing models of healthcare provision.

## Conclusions

Screening asymptomatic individuals for dementia raises complex issues and a number of factors impact on its acceptability. For a few delegates, screening was seen as a potentially positive experience; however, for others the reverse was true. In this event the general public had a largely sceptical view of the benefit and validity of population screening for dementia. Psychological and physical health of patients, taboos about dementia, a number of practical issues and, crucially, the perceived lack of benefit need to be addressed before screening would be acceptable to the population. Findings also demonstrate a level of uncertainty about what dementia is, how it develops, methods of identification, the prognosis and treatment of the syndrome. It is unclear which interventions are required to change the perspectives of the general public. Future studies can utilise the information contained in this investigation of public opinion to inform a larger qualitative research project that may answer these questions.

## Additional file

Additional file 1: Table S1. Themes identified in studies. (DOCX 42 kb)

## Abbreviations

ALCOVE: Alzheimer’s Cooperative Valuation in Europe
PPI: Patient and Public Involvement
GRIPP: Guidance for Reporting Involvement of Patients and Public
ASRN: Alzheimer’s Society Research Network
NHS: National Health Service
