# Supplementary material for: Exploring attitudes and preferences for dementia screening in Britain: contributions from carers and the general public
Source: BMC Geriatr. 2015 Sep 9;15:110. doi: 10.1186/s12877-015-0100-6 (PMC4564978; doi:10.1186/s12877-015-0100-6)
Supplement: Additional file 1: Table S1. — Themes identified in studies. (DOCX 42 kb) [file 12877_2015_100_MOESM1_ESM.docx]

**Supplementary table 1: Themes identified in studies**

| **Pre-screen** | |
| --- | --- |
| **Theme** | **Evidence** |
| **Accuracy of the test** | “I’d want to know how effective it is... I’d want to be given some figures”; “Yeah, the accuracy of the test is everything. At the moment it’s our considered opinion that the test is in its infancy”.  “But what are you going to screen for? In bowel screening and in breast screening it’s quite clearly known what you’re searching for and it’s quite clearly known what you’re going to do if you find it. My first question is what are you going to screen for?”  “I don’t think they can ever guarantee that for any medical procedure, but correct me if I am wrong. I don’t think there is ever no risk in a procedure”.  “The problem with the testing is that you want to ask questions that are related to themselves personally. Their memory loss, whether they remember it or not, it’s too generic to use as a testing format. However, to ask questions to a memory that may not be related to them in any way, maybe too foreign, the way it’s tested now is... but if you want to test for memory loss, memory loss itself is something that’s so hard to measure and is so subjective that to standardise a form of test seems invalid”.  “There is research that could be done that would lead to then screening to validate the research, but they don’t seem to be in that position at the moment, but they might be just getting there”.  “But the screening... well we haven’t seen it, but the screening seems to be so loose that if you go on that clinical presentation and you put it in front of a bunch of medics that really understand, there’s probably dozens of different diagnoses that could be made. An awful lot of them are treatable, and so the person might have been diagnosed with something else in the interim that will account for whatever this positive screening was, and now they’ve been diagnosed and treated with it, well they don’t think they need to participate any more”.  “That’s the problem, when it’s so loose, imprecise”.  “Until there’s a good test and something to be offered afterward, you know, a sort of accurate test and the possibility of knowing what’s going to be offered afterwards, then there is no point”.  “So it needs to be a reliable test and then something positive following that. Because if the test is unreliable, even if you’ve got a positive follow on, you’ve got to get it for the right people. It’s like clinical trials, until they can divide up the dementias and give the drug to the people with the same condition well their clinical trials are going to fail. So you might have a positive outcome, but it won’t come across as that if we don’t get a sound test as well” |
| **Existing care** | “They make you wait for certain things”.  “A lot of the staff members… treated them (patients) like animals”.  “I mean if you are in the system already you’re much more likely to see the publicity about do you want to join... you know, sign up in the thing, you know, you’re more... or you’re in the hospital you see a sign and think oh yes... perhaps I should be doing that!” |
| **Existing health state** | “You’ve already been diagnosed with something and they’re helping, and you’re being treated, so there is a cause and an answer which would create more confidence”.  “Worry more”. |
| **Experience with disease** | There was no consensus between those with experience of the disease and those without. Some, with experience, argued, “I have a few acquaintances who have dementia, and it makes it much more real”. These were more likely to accept screening. However, some had family members with the disease and were dissuaded from undertaking the screen because of this experience: “Having someone in the family or close with a condition does not make you any more likely to seek answers for it”.  Some argued that previous experience had no bearing on what is perceived as an individual choice: “Having someone in the family, I wouldn’t say affects you at all, affects your likelihood of going for screening at all”. |
| **Financial motive** | “Somebody could make money”;  “Push on drugs”.  “I would have suspicions if it was being done by a drug company”.  “The media selling it (dementia) as a fear tactic”.  “Risk then that I could be clobbered for higher insurance or not get insurance”.  “Yeah, it’s expensive, but years ago they were done by the county council, they used to have social services, used to have their carers, home helps, etc. etc. Now it is done on a commercial basis, on commercial terms, which are getting a profit out of it, and the county council are supposed to be paying less, there would be more money if they employed their own staff… it has been done on a cut price basis, and the people they get in to do it, they get whoever they can, on basic legal minimum wage… and very little training”. |
| **Lifestyle and life view** | “If you’re going to risk losing your job… losing whatever… then you’ll probably back off until there is a precise treatment”.  “Get on with your family life”.  “It’s the balance... of what you have to go through for the screening... what the benefits are, you get a return, or carers get a return, it’s that sort of balance”.  “Yeah, exactly, it’s a personal choice, and I think if you know that your lifestyle could lead to dementia you’d probably be more comfortable with your lifestyle and not want to worry about dementia.” |
| **Other screening experience** | “Means you go and get your boobs squashed”.  “Is very invasive of your body”.  “That’s completely different, the way you think about it and anticipate it”. |
| **Role of clinician** | “My daughter (is) qualified (as doctor), she was treating people… going to discharge them and saying has their dementia been managed… and consultants were saying to her ‘Well, they’re not diagnosed with dementia’… She’s always trying to get them care plans and fighting for their support, but you know, while it’s not even known widely at that level amongst the professionals, then I think there’s a lot of work to be done, and not just in the community”. |
| **Role of culture** | “I was talking to a girl in one of the other groups, an Indian girl, about extended families, and in India as well it is not a problem, because you have people around”.  “My wife is Chilean, and when we go over there it is a family concern over there, they really do look after each other”.  “I live in the Philippines most of the year and I have a big extended family, and they look after me… and it is one advantage you have in third world countries, they have extended families… there is no suffering”. |
| **Role of family** | “Her husband refused to take her”.  “Was upsetting and worrying for her (the mother-in-law), she knows what Alzheimer’s is, knows that it presents in different ways and it was terribly distressing, and it would be distressing if your family suggested that just because you keep losing your keys or you can’t remember”.  “A lot of people tend to keep it in the family, than let outsiders know that it is happening, don’t they? That is the problem.”  “It sounds dreadful but in fact, it is true isn’t it, for a family to have to learn that some member of the family is going to come down… be felled by this disease is just awful. You’re living on a knife edge and borrowed time”.  “I think in a lot of cases it would be people being persuaded by their partners”.  “I think this screening is just as important for the family members as it is for patients, to get an idea of what can be expected, to be a little more informed, especially of the psychological aspects and to be more prepared themselves physiologically as well, I think it is important from that view”. |
| **Who to target?** | “School”.  “The immediate problem surely is how are you going to get younger people to take the test”.  “40 and upwards”.  “You should target everyone rather than a certain group of people like based on age or ethnicity or experience or whatever”.  “Higher risk groups should be sorted out initially and need to be screened first”.  “I think you should target everyone rather than a certain group of people like based on age or ethnicity or experience or whatever”; “They should do that for the population, all the population”; “25 years old to 75”; “Anyone with no symptoms at all”; “People in their twenties”.  “Well, everything prior to this, why would people want us to take a test if we are asymptomatic, if we are asymptomatic then there is no need”. |
| **In-screen** | |
| **Theme** | **Evidence** |
| **How to test** | “I’d still be interested how it would be administered, you know”.  “Would brain scans be appropriate for a screening”.  “The form of screening that I would opt for, if there was a choice, would be to go and have the head scan because it shows whether you’ve got Parkinson’s or Alzheimer’s. I mean, I do know that because that’s how we found out my mother had got it, and my uncle, and what they’d got, because green, I think it’s green, or was it blue, is the Alzheimer’s, red is the Parkinson’s”. |
| **Learning of test** | “Yeah, the woman actually went in there and she did a test, a memory test with my mother, and my mother already previously knew that this woman was coming because they have to tell them, and I went, and I was sitting there and I’m thinking... well, you’re asking her questions that she’s already logged in her head and she knows, and she’s going to reel them off, as she was doing”. |
| **Organisational pressures** | “The doctor doesn’t have time, you know, you rush in, you’ve got five minutes, and away you go, so if you have two ailments and you have two questions you have to get another appointment, so you know, why is this doctor doing the screening?”  “It would put more pressure on the GPs because they would have to have training, which means more closed surgery days, and they will have to have special times to do that, you can’t do it on a normal ten minute appointment. So yeah, that would put pressure on them to be honest”. |
| **Relationship to doctor and health** | “I think if you’ve got a good rapport with your doctor, you feel comfortable, and probably with a doctor you’re familiar with, they spend a bit more time with you than a doctor you don’t know, so I think that probably... well, in my case it would make it easier”.  “If you have a known treatable condition that has been diagnosed, and you are receiving treatment, that might build your confidence in the medical profession and medical practice and you might be more likely to go and agree to be screened, thinking that they wouldn’t do it unless they had something that they could do for you”.  “Even if you just visit regularly for different things, and if you have the same GP you see all the time, if they come and suggest this screening… you might be more inclined to accept it. Because otherwise, if you see a different GP every time and one of them offers it to you, you say no, I’ll pass. I think that, yeah”.  “I mean if you have two illnesses you’re much more likely to have a rapport with your clinician aren’t you? If you’re fit and healthy some people never go anywhere near a doctor for years on end, so you wouldn’t have that”.  “I think people have too much of a distaste for the NHS. My two views are slightly questionable. My personal view is that the NHS leaves everyone with a bitter taste in their mouth, national distrust, like the media has had enough time of their inadequacies, and splashing the bad stuff all over the news and they have brought it on themselves a lot”.  “The NHS has saved my life several times” |
| **Training needs for staff** | “I used to work as a labourer for a BUPA care home, and there was a lot of people in there with dementia, and no disrespect, I put it down to a lot of the staff members, the way they treated them, because they treated them like animals, so they did, and I mean like animals”.  “Well there’s been stories about it haven’t there, in the paper, on the news, you know, and it’s like how can one human treat another human like that, because it’s not right. So yeah, something needs to... something in that aspect needs to be done as well for certain things”.  “That’s training isn’t it? People administering the tests are not trained in any way”.  “Well they need to be trained, it’s got to be someone who’s trained... and it’s not going to be achieved”.  “It’s got to be somebody trained to do that and not anything else”.  “Yes, we’re looking for the good test that a trained person can administer”. |
| **Who should conduct the screen?** | “It’s got to be somebody who’s trained, who knows what to look for, who knows what care they’re actually dealing with ...”  “I think as a general practitioner they get five minutes with you, and to make decisions you know... so maybe you’re better off with a more specialist... somebody who deals with this”.  “Maybe an independent body or something”.  “I mean most surgeries have a CPN or others actually, don’t they in most surgeries have other people involved, presumably they could be passed on to the appropriate person to do the actual screening rather than the GP”.  “The first step would be done through your main carer, health carer”.  “A carer that does a test”.  “I would prefer a doctor, my own GP”.  “It could be a memory clinic that’s doing it, it could be a department of the hospital”.  “Well, have mobile vans coming round!”  “Social Services already use a test to test people for dementia in the nursing homes”.  “Family, carers, nurses”. |
| **Post-screen** | |
| **Theme** | **Evidence** |
| **Planning** | “Rather know to be prepared, you know, set things in place before you are in a state where you can’t remember anything”.  “For me not to do with jobs or insurance… just lifestyle”.  “Don’t know whether I’m going to end up in a wheelchair or not”. |
| **Role of support** | “You can’t cure it, but if there’d have been some help”.  “Would want to live my life and not to worry, but if I was diagnosed with dementia and I had the right support around me, fair enough I’d like to do things”.  “Would want to live my life and not to worry, but if I was diagnosed with dementia and I had the right support around me, fair enough I’d like to do things”.  “Getting them involved in providing a good support network for each other, to be there for each other as well as the person affected, and I think it probably just wakens people up to just sort of try and have a better quality of life rather than dwell on problems and depression and bad weather”.  “But you can get all of that without screening”.  “Screening cannot interfere with my life. As a woman I have many different roles in my home, looking at my family and husband and depending on what is happening, I might not have time to go for screening”. |
| **Social impact** | “I think all that might do is just engender tremendous anxiety in the population at large”.  “I think we think it could be detrimental and it’ also... unnecessary and the cost involved would be just...”  “One of the things that would put me off I think is like... maybe you test positive… what if that (information) gets into the wrong hands, like employers or like... that can affect you too, like ‘Oh well we won’t employ that group because...’ Someone’s got information somewhere about you which is possibly... that’s what would put me off, that would be a negative”.  “But if you’re going to risk losing your job, if you’re going to risk losing whatever in your current lifestyle, then you’ll probably back off until there is a precise treatment”. |
| **The screen result** | No consensus. Some wanted to know more (“You should find out for the sake of it”) while others preferred not to know, “a fear of knowledge”.  “Waiting for the result I think is the hardest part”.  “I am not sure knowing actually benefits me”.  “If you’re the kind of person who hides from the truth and reality, you’re not going to be any more likely to go”.  “You may not be apprehensive of the test itself, but certainly apprehension of the results”.  “I want to know the implications of the results”.  “It’s demoralisation”.    “Given a death sentence”.  “I think it’s a bit more than a death sentence as well”.  “You slowly drown into pretty much a vegetable”.  “If I discover I have dementia then Jesus! My whole life and perception will change and try and shape things the other way around”.  “I mean, excuse my bluntness but if someone finds out you have dementia at quite a young age… that you will be completely forgetful and a drooling mess… a vegetable and we don’t want to be that and you don’t want to see yourself… having slow degradation of quality of life”.  “I personally don’t think I’d want to know, I wouldn’t want to be told that I’d got it or if I have or haven’t got it because I just think it’d just cause more stress in your own mind”; “I’d rather not know, I’d rather just get on with life, if something’s going to happen it’s going to happen”.  “I’d definitely want to know. I’ve seen it with my father, and my mother trying to hide it from everybody, she was frightened to death of what people would say, and even neighbours she tried to hide it from them, and you know it was ridiculous really, but she just wouldn’t get advice or help: ‘I can cope, I can cope’”.  “There are enough problems, concerns about growing older anyway, and if you suddenly decide, suddenly learn, that you are going to be invaded by this horrible condition, then what, you’re better not… The doctor said to my sister who died of lung cancer, well, we didn’t get in touch with you as early as we may have done, but just think: you had nine months where you didn’t have to worry because you didn’t know you got it. Well, it sounds dreadful but in fact, it is true isn’t it, for a family to have to learn that some member of the family is going to come down, be felled by this disease, is just awful. You’re living on a knife edge and borrowed time”. |
| **Themes which cut across the pre-, in-, and post-screen process** | |
| ***Acceptance of test***  Some respondents had undertaken the MMSE and noted that the test itself is “stressful”.  “I wouldn’t want to be screened… if there was any side effects or if the test got too intrusive”.  “How do we then define a simple test, because there are some very simple tests which if you’re not interested in what they’re asking about you will forget about it, you will not even remember to answer him properly, but if it is something that you like and they test you on that you remember…”  “If you said a simple written test, it’s not a simple written test for anyone who can’t write... so a simple written test will eliminate quite a fair number of people”.  “Yeah but unfortunately to explain to someone to go for testing they usually send you a piece of paper with a lot of writing on it, so those sort of things do eliminate people from the start”.  “I think the information comes after the test, that’s the thing, because very few people would sit down and say ‘I really want to know’, they just want...”  “Yeah, the actual nature of the test has got to be known beforehand, you’ve got know what you’re letting yourself in for, otherwise you might not even walk through the door. But if you know that you could do this test in half an hour, ten minutes, whatever it happens to be and you get the result the next day...”  “You need to get up to a certain level for it to achieve anything, so would screening be the same or would it be ‘Oh well, if you don’t want to do it fine’... You know, I mean the thing is... well if 20% got screened and the rest didn’t, I mean is there any point?”  “It was very stressful, and I sat there, I was terrified I wouldn’t get 30 out of 30, and I found it very stressful, and I think probably just doing these tests is actually quite stressful because there’s a feeling that you might fail for heaven’s sake, you know”.  “I always think the problem with tests is that different people have different attitudes. Some people the mere thought of sitting a test makes them nervous, so they will underperform, and in a test like this it’s a subjective test isn’t it” | |
| ***Awareness of disease***  “Alzheimer’s is one of these nebulous, I am not really sure what it is”.  “Alzheimer’s… there is somewhere around 100 different forms of Alzheimer’s”.  “I would just like to know how far science or knowledge has gone with regards to understanding Alzheimer’s or dementia, are they two words for the same thing?”  “Maybe it could be down to stress, maybe it could be... I don’t know... fertilizer they put on food”.    “There must be an inheritance factor”.  “Some say it’s the scrapings off the pot, when you’re cooking you’re using a grey aluminium pot”.  “I think it’s the foundation is on how much we currently know on dementia, or in other words, how much does the medical system know?”  “Screen (with) adverts on the TV you’ll change peoples’ perception of it (dementia)”.  “I feel uncomfortable... I feel uncomfortable discussing it”.  “Because it’s similar, the signs of aging are similar to dementia, just growing old and forgetful”.    “I am not sure what the difference between dementia and senility is, I mean as people get older they do just deteriorate a wee bit”.  “Guinea pig”. | |
| ***Costs***  “If there is no positive implications (no cure), is it worth spending the money”; “That’s an awful lot of money in terms of paying the doctors and time taken off their work”; “It would probably be very expensive”; “It could be better spent”; “Well it’s a waste of money”; “There is no funding for this”; “I mean unless they’re going to put money into the system it’s actually fairly pointless”.  “There’s more and more money being taken out of the NHS, and this system, screening, is requiring more money not fewer doctors and nurses and care workers. So why... forget it, put the money into research. Forget the screening”. | |
| ***Lack of ability to change prognosis***  “I’d still rather be in my house where I’ve got a familiar environment to me, and I know where... especially if I know my brain is deteriorating, the last thing I’d want is to be surrounded by strangers in a completely different environment and everything’s in a set regime and all that sort of stuff. So I think yeah, a care home is probably the last place I’d want to be”.  “I think care homes are like regimental, but on the other hand I reckon they design them like that so the people that have got dementia… (have) got a set routine, like breakfast at nine, lunch at 12, tea at half five, and then supper at like eight… so they’ve only got to remember limited things. That’s a way of helping them cope with it”.  “If there was a cure or they could head off that disease, that would be something to encourage you for it, for a test”.  “Why isn’t there a cure for it though?”  “I’d probably change my opinion on it if I knew for certain there was actually a treatment for it that worked. But at the moment I just think there’s so much research, but there’s no treatment for it”; “I think you’re better not knowing nothing about it at all, personally speaking I’d rather not know at all, but I... I could be influenced”.  “You’re still facing the possibility of being told that you might have a disease for which there is no cure”.  “You can't be cured anyway, my guess is. Yes have another glass and forget it all”.  “But it depends if there is treatment, there is no treatment for Alzheimer’s, there is no treatment for lots of these neurological diseases, no treatment for Parkinson’s, there is nothing you can do”.  “Side effects… That’s something I wrote down because whether they have to do it to my body, whether that’s a medical condition and all of that, I have to be very careful about not having side effects. If it’s a question of just pen and paper method that’s fine, but when it gets to the next stage of further treatment, I would be very, very shaky, and like is this going to affect me in any way… You are going to have to give me a complete guarantee that nothing is going to happen and I am going to be very careful in terms of side effects”.  “I must say I haven’t felt like doing it, but I could understand... I could actually completely understand... and I must say if I thought I’d got the first signs of dementia I don’t know… I think I’d start hoarding pills. I should start doing something because it... well, you know…”  “They’re also assessing the anti-psychotics, which is good, you know, that’s fundamentally positive”.  “There is no treatment apart from palliatives”. | |
| ***Patient Benefit***  “From what we know right now … I am not sure knowing actually benefits me”; “Dementia, it’s one of those ones where if you get it there’s not much you can do about it, so unless I had specific reasons to do it I don’t think I’d have it”; “I wouldn’t have the stress of worrying about it”.  “I just don’t understand why you wouldn’t go”.  “(They) see it as quite positive really, I think I am lucky to live in a country where there is this sort of screen for this disease”.  “It is my choice, so I would be informed about it beforehand and then I would make my choice, so I don’t see a problem there”.  “You can’t change what’s going to happen and what’s not going to happen”  “If you knew that your husband was going to get dementia. If you had known five years before he was finally diagnosed, would it have made life any different?” The respondent replied: “No, it wouldn’t have made any difference”. | |
| ***Stigma***  “I think there’s a sort of stigma attached to going for screening, and people will be ducking and diving, you know, so I think it’s important”; “If you are not careful you are going to be labelled”; “Because of the stigma”; “Rightly or wrongly, it has a stigma”.  “People (would be) judging you for an illness (and this) shouldn’t really be right”; “Too many of the population have a stigma, you know, and... just because they don’t know how to handle it in my view”.  “I’ve been a carer, and you have ten minutes with that patient, they’re a human being for god’s sake”.  “Remember a thing on TV though, right, it’s always sort of wealthy people what they’re showing... they don’t show the man in the council flat do they... Why is that?”; “Every programme I’ve ever watched... they do tend to show the upper class people most of the time”.  “The stigma of mental health and everything, you know, now it’s not so much stigmatised, you know”. | |
